# Use of Botulinum Toxin for Esotropia Treatment in Government Hospitals of Bahrain: A Cross-Sectional Study

**DOI:** 10.7759/cureus.105338

**Published:** 2026-03-16

**Authors:** Hessa M Alamari, Yusuf A Bucheery

**Affiliations:** 1 Department of Ophthalmology, Salmaniya Medical Complex, Manama, BHR

**Keywords:** botulinum toxins, esotropia, exotropia, ophthalmology, strabismus

## Abstract

Background: Strabismus is a common pediatric ophthalmologic disorder characterized by misalignment of one or both eyes that is managed by surgical and non-surgical treatment options. Botulinum toxin has emerged as an alternative to surgery for certain types of strabismus. However, limited data exist regarding its use and outcomes among cases of strabismus in Bahrain. Therefore, this study aimed to evaluate the characteristics and outcomes of botulinum toxin treatment in patients with strabismus at government hospitals in Bahrain.

Methods: A cross-sectional study was conducted in ophthalmology clinics in government hospitals in Bahrain. All patients with strabismus who received botulinum toxin were included. Demographic characteristics, strabismus type, dosage, treatment outcomes, and complications were collected. Descriptive and inferential analyses were performed.

Results: The study included 31 patients with a mean age of 6.3±4.3 years; 16 (51.6%) were female. The most common strabismus type was partially accommodative esotropia (PAET) 16 (51.6%). A single injection was administered to 21 (67.7%) patients, with 11 (35.5%) achieving orthotropia after the first dose. After the injections, orthotropia and ptosis were noted in 17 (54.8%) and 14 (45.2%) of the cases, respectively. A significant association was found between orthotropia after the first dose and the occurrence of exotropia (p=0.026). Higher preoperative angles were significantly associated with higher initial botulinum toxin doses (p <0.001). Surgical intervention was required in seven (22.6%) cases.

Conclusion: Botulinum toxin is an effective treatment for strabismus cases in Bahrain with a relatively low rate of complications. Extending its use in cases of strabismus might reduce the need for surgical intervention and improve the overall care for patients with strabismus.

## Introduction

Strabismus is a prevalent ophthalmological disorder in children characterized by misalignment of one or both eyes [1]. Depending on the direction of eye deviation, strabismus is classified into different types: esotropia, the inward deviation of the eyes; exotropia, the outward deviation of the eyes; hypertropia, the upward deviation of the eyes; and hypotropia, the downward deviation of the eyes [2]. The prevalence of this condition varies across the studies. A systematic review of 56 articles that included nearly 250,000 patients revealed a prevalence of 1.93% of all types of strabismus, with a higher prevalence of exotropia (1.23%) compared to esotropia (0.77%) [3]. In Saudi Arabia, the prevalence of strabismus was around 0.5% among school children [4,5].

Although the treatment of strabismus depends on the cause, it generally includes a combination of nonsurgical and surgical interventions aimed at improving ocular alignment and vision. Non-surgical management involves observation or interventions such as spectacles, prism therapy, miotic drops, occlusion, and visual training. Surgical options like extraocular muscle recession and resection are used to correct misalignment, with a generally rapid recovery. Botulinum toxin has also been used to treat strabismus. Commonly called Botox, it is an injectable medication that blocks the nerve signals, resulting in temporary paralysis of muscles. Several forms of botulinum toxin, including onabotulinumtoxin-A (Botox), abobotulinumtoxin-A (Dysport), and incobotulinumtoxin-A (Xeomin), have been used to treat strabismus [6]. The success rates of botulinum toxin in treating strabismus range between 50% and 95% [7,8]. According to the literature, the success rate increased with subsequent injections. Additionally, the studies reported that transient exotropia (57.14%) and transient ptosis (49.20%) are common adverse events among those who underwent botulinum toxin injection therapy. Moreover, up to 20% of patients eventually required surgical intervention [9].

A systematic review of six interventional studies found variable results for botulinum toxin in addressing strabismus, with minimal impact on abducens nerve palsy and horizontal strabismus [10]. Compared to surgery, some studies reported that botulinum toxin is more effective in treating strabismus [11]. In comparison, other studies found a lower success rate with botulinum toxin when compared with surgical interventions, especially in horizontal strabismus. A study among 56 children with esotropia reported high success rates (75%) even with the low dose (2.5 IU), with ptosis affecting up to 37.5% of patients [12]. A study of 86 patients reported varied success rates depending on the type of strabismus, with the highest rates noted in Duane retraction syndrome (75%) and residual esotropia (61.5%), while the lowest rates were noted in sixth nerve palsy (13.3%). The study also revealed no correlation between success and age, sex, or dose, and no association between side effects like ptosis and dose-related ones, while early overcorrection was the single strong predictor of success rate [13]. Another study among 42 patients with acquired non-accommodative comitant esotropia revealed success rates of 90.5% at six months, 76.2% at one year, and 73.8% at final follow-up, with baseline esodeviation being the only significant predictor of outcome [14]. In a study of 403 children with esotropia treated by botulinum toxins, the success rate reached 47.4%, with higher rates noted in partially accommodative esotropia (53.1%) compared to infantile esotropia (37.1%). The study also showed that a smaller baseline deviation and early overcorrection were significantly associated with better outcomes (p < 0.001), while side effects included transient overcorrection (63.8%) and ptosis (41.7%) [15].

Although few studies were conducted to assess the characteristics and outcomes of botulinum toxin use in treating strabismus, no previous studies were conducted to determine their efficacy and outcomes in Bahrain. Therefore, this study aimed to evaluate the characteristics and outcomes of botulinum toxin treatment in patients with strabismus at government hospitals in Bahrain.

## Materials and methods

Study design

An observational study was conducted in a specialized ophthalmology clinic in a government hospital in Bahrain to evaluate the outcomes of botulinum toxin treatment in patients with esotropia. The study included patients with esotropia who received botulinum toxin treatment in government hospitals in Bahrain between 2019 and 2023. Ethical approval was obtained from the Research and Research Ethics Committee for Government Hospitals, Bahrain (approval no. 40-240425 dated 24th April 2025). All patients with esotropia who were treated with botulinum toxin in government hospitals were included in the present study. There were no exclusions based on age, sex, or medical history.

Procedures and post-treatment assessments

Patients underwent a detailed ophthalmic examination, including visual acuity measurements, cycloplegic refraction, and anterior and posterior segment examination. A complete orthoptic examination was performed by a single pediatric ophthalmology and adult strabismus consultant. The angles of deviation in the primary position at a distance of 33 cm and 6 m were measured with an alternate prism cover test after the full optical correction. The Krimsky method [16] was used for patients who were unable to fixate due to low visual acuity or for infants for whom only the angle at near was measured, as they could not fixate at a distant target.

Next, 2 mL of balanced salt solution was injected into the bottle containing 100 IU of botulinum toxin to obtain 5 IU per 0.1 mL. Then, the botulinum toxin was given using a 27-30-gauge needle in different dosages (2.5, 5, 7.5, and 10 IU) according to the respective size of deviation (11-19, 20-29, 30-39, and ≥40 prism diopters (PD)).

Post-treatment measurements were performed at two weeks, three months, and six months after the injection. Reinjection of botulinum toxin was considered at six months after the initial injection if the angle of esodeviation was >10 PD during distant fixation. Patients with a follow-up duration of at least 12 months after their last botulinum toxin injection were included for analysis. A successful outcome was defined as deviation ≤10 PD in the last visit (a minimum of 12 months) following the injection.

All injections were performed under sedation, and topical anesthesia was administered by the same consultant ophthalmologist. Visualization of the anterior ciliary vessels and the medial rectus from under the conjunctiva facilitated accurate injection, as it was given blindly without electromyogram (EMG) guidance or direct visualization of the muscles.

Data collection and analysis

The data collection tool included patient characteristics such as age, sex, presence of medical conditions, and type of esotropia; treatment characteristics such as number of injections received, post-treatment ptosis, and angle characteristics; and outcomes such as the need for additional surgeries. The collected data were organized in a Microsoft Excel sheet (Microsoft Corp., Redmond, WA, USA) and then transferred to SPSS Statistics version 27 (IBM Corp., Armonk, NY, USA) for statistical analysis. Continuous variables were expressed as means with standard deviations, while categorical variables were presented as frequencies and percentages. The Shapiro-Wilk test [17] was used to test the normality of the data. Fisher's exact test was used to determine differences between groups. A p-value of less than 0.05 was considered statistically significant.

## Results

A total of 31 patients were included in this study, with an average age of 6.3 ± 4.3 years. Among them, 14 (45.2%) were under five years old, 11 (35.5%) were between five and 10 years old, and six (19.4%) were older than 10 years. A pre-existing medical problem was identified in 12 (38.7%) of the patients, and the most prevalent type of esotropia was PAET in 16 (51.6%) patients. Table 1 presents the demographic and clinical characteristics of the patients.

**Table 1:** Demographic and clinical characteristics of patients (total n = 31) PAET: Partially accommodative esotropia; AET: Accommodative esotropia

Variables	Categories	n (%)
Age	<5 years	14 (45.2)
	5-10 years	11 (35.5)
	>10 years	6 (19.4)
	Mean ± SD	6.3 ± 4.3
Gender	Male	15 (48.4)
	Female	16 (51.6)
Medical problem	No	19 (61.3)
	Yes	12 (38.7)
Type	PAET	16 (51.6)
	Infantile	7 (22.6)
	Other non-accommodative esotropia (AET)	8 (25.8)
Preoperative angle	11-19	1 (3.2)
	20-29	10 (32.3)
	30-39	11 (35.5)
	≥40	9 (29)
Injection numbers	One injection	21 (67.7)
	Two injections	9 (29)
	Three injections	1 (3.2)

For the first injection, almost half of the patients (n=15, 48.4%) received 10 units of botulinum toxin, followed by five units (n=8, 25.8%). Post-first dose, approximately one-third of patients (n=11, 35.5%) achieved orthotropia, while the majority did not (n=20, 64.5%). Ptosis was observed in n=11 (35.5%) cases, while exotropia occurred in n=16 (51.6%). A second dose was administered to 10 (32.3%) patients. Of all those who received a second dose, most (n=6, 60%) received 10 units, and half of them (n=5, 50%) achieved orthotropia, while four (40%) remained non-orthotropic. Ptosis occurred in two (20%) of the patients, and exotropia was observed in four (40%). Only one patient required a third dose (10 units), which resulted in orthotropia (n=1, 100%) but was associated with ptosis and exotropia. After all injections, orthotropia and ptosis were noted in 17 (54.8%) and 14 (45.2%) cases, respectively. Table 2 shows the number of doses given and their initial outcomes.

**Table 2:** Doses given to patients and their initial outcomes (total n = 31)

Variables	Categories	n (%)
First dose (total n =31)	2.5 IU	2 (6.5)
	5 IU	8 (25.8)
	7.5 IU	6 (19.4)
	10 IU	15 (48.4)
First dose angle	Non-orthotropic	20 (64.5)
	Orthotropic	11 (35.5)
Ptosis first dose	No	20 (64.5)
	Yes	11 (35.5)
Exotropia first dose	No	15 (48.4)
	Yes	16 (51.6)
Second dose (total n=10)	2.5 IU	1 (10)
	5 IU	2 (20)
	7.5 IU	1 (10)
	10 IU	6 (60)
Second dose angle	Non-orthotropic	4 (40)
	Orthotropic	5 (50)
Ptosis second dose	No	8 (80)
	Yes	2 (20)
Exotropia second dose	No	6 (60)
	Yes	4 (40)
Third dose (total n =1)	10 IU	1 (100)
Third dose angle	Orthotropic	1 (100)
Ptosis third dose	Yes	1 (100)
Exotropia third dose	Yes	1 (3.2)
All doses angle (total n=31)	Non-orthotropic	14 (45.2)
	Orthotropic	17 (54.8)
Ptosis after all doses (total n=31)	No	17 (54.8)
	Yes	14 (45.2)
Surgery (total n=31)	No	24 (77.4)
	Yes	7 (22.6)

As shown in Table 3, the analysis of the relationship between first-dose response and patient characteristics showed no significant correlation with strabismus type (p=0.787). However, a significant association was found between exotropia and the first-dose angle (p=0.026), suggesting that exotropia is more likely to occur in patients achieving orthotropia after the first dose. There was no significant association between first-dose response and ptosis (p=0.238). This data is illustrated in Table 4.

**Table 3:** Relationship between the first dose angle, type, and preoperative angle The p-values were computed using the Fisher-Freeman-Halton exact test. PAET: Partially accommodative esotropia, AET: Accommodative esotropia

Variables	Categories	First dose angle		Fisher-Freeman-Halton exact value	p-value
		Non-orthotropic	Orthotropic		
		n (%)	n (%)		
Type	PAET	9 (56.3)	7 (43.8)	0.864	0.787
	Infantile	4 (66.7)	2 (33.3)		
	Other Non-AET	6 (75)	2 (25)		
Preoperative angle	11-19	0 (0)	1 (100)	2.670	0.517
	20-29	5 (55.6)	4 (44.4)		
	30-39	7 (63.6)	4 (36.4)		
	≥40	7 (77.8)	2 (22.2)		

**Table 4:** Relationship between preoperative angle, the number of injections, and first dose The p-values were computed using Fisher’s exact test; *p-value significant at 0.05

Variables	Categories	First dose angle		p-value
		Non-orthotropic	Orthotropic	
		n (%)	n (%)	
Ptosis first dose	No	14 (73.7)	5 (45.5)	0.238
	Yes	5 (26.3)	6 (54.5)	
Exotropia first dose	No	12 (63.2)	2 (18.2)	0.026*
	Yes	7 (36.8)	9 (81.8)	

The preoperative angle showed a significant relationship with the initial botulinum toxin dose (p <0.001), indicating a trend of higher doses being administered to patients with larger angles of deviation. However, the number of injections required was not significantly associated with the preoperative angle (p=0.236). No significant relationships were found between second-dose angle and ptosis (p=0.444) or exotropia (p=0.524). Tables 5-6 highlight these results. As illustrated in Table 7, surgical intervention was required in seven cases (22.6%). But the presence of a medical problem and preoperative angle were not predictive of the need for surgery (p=0.676 and p=0.661, respectively).

**Table 5:** Relationship between preoperative angle, number of injections, and first dose The p-values were computed using the Fisher-Freeman-Halton exact test; *p-value significant at 0.01

Variables	Categories	Preoperative angle				Fisher-Freeman-Halton exact value	p-value
		11-19	20-29	30-39	≥40		
		n (%)	n (%)	n (%)	n (%)		
Numbers of injections	One injection	1 (100)	9 (90)	7 (63.6)	4 (44.4)	7.952	0.236
	Two injections	0 (0)	1 (10)	4 (36.4)	4 (44.4)		
	Three injections	0 (0)	0 (0)	0 (0)	1 (11.1)		
First dose	2.5 IU	1 (100)	1 (10)	0 (0)	0 (0)	29.828	<0.001**
	5 IU	0 (0)	7 (70)	1 (9.1)	0 (0)		
	7.5 IU	0 (0)	2 (20)	4 (36.4)	0 (0)		
	10 IU	0 (0)	0 (0)	6 (54.5)	9 (100)		

**Table 6:** Relationship between the second dose angle, ptosis second dose, and exotropia second dose The p-values were computed by using Fisher’s Exact Test.

Variables	Categories	Second dose angle		p-value
		Non-orthotropic	Orthotropic	
		n (%)	n (%)	
Ptosis second dose	No	4 (100)	3 (60)	0.444
	Yes	0 (0)	2 (40)	
Exotropia second dose	No	3 (75)	2 (40)	0.524
	Yes	1 (25)	3 (60)	

**Table 7:** Relationship between surgery, medical problem, and preoperative angle ^a^The p-values were computed using Fisher’s exact test; ^b^The p-values were computed using the Fisher-Freeman-Halton exact test

Variables		Categories	Surgery			p-value
			No	Yes	Test value	
			n (%)	n (%)		
Medical problems	No		14 (73.7)	5 (26.3)	Not applicable	0.676^a^
	Yes		10 (83.3)	2 (16.7)		
Preoperative angle	11-19		1 (100)	0 (0)	2.141	0.661^b^
	20-29		9 (90)	1 (10)		
	30-39		8 (72.7)	3 (27.3)		
	≥40		6 (66.7)	3 (33.3)		

## Discussion

The study aimed to assess the outcomes of botulinum toxin treatment for esotropia in patients attending ophthalmology clinics in government hospitals in Bahrain. The results show that most patients required only one injection, while only 22.6% of patients eventually required surgical intervention. In addition, orthotropia was noted in more than one-third of patients following the first dose of botulinum toxin and an additional 16.1% after a second dose.

In line with previous literature, this study shows that botulinum toxin was efficacious in treating esotropia. A study conducted in Saudi Arabia on infantile esotropia reported a 51% success rate with botulinum toxin injections, with improved outcomes after repeated doses [9]. In the current study, a similar pattern was observed, as the total orthotropia rate increased with additional injections. International reviews have shown motor alignment success rates of approximately 60% with botulinum toxin, comparable to those achieved with surgery in certain cases. Our findings support these results, as a combined total of 54.8% (17/31) of patients achieved orthotropia after one or more doses. This aligns with evidence suggesting botulinum toxin can be a viable non-surgical alternative, particularly in early or less severe cases. Nonetheless, higher success rates (>90% in some studies) are also reported in the literature. The variation in botulinum toxin success rates is attributed to differences in strabismus type, baseline deviation size, number of injections, and dosages.

In terms of side effects, the most frequently observed complications were transient ptosis and exotropia. In this study, ptosis was noted in 35.5% of patients after the first dose and in 6.5% after the second dose, while exotropia was observed in 51.6% following the first dose and in 12.9% after the second. These rates are comparable to previous studies, such as the one from Saudi Arabia that reported transient ptosis in 49.2% and exotropia in 57.14% of patients [9]. Other reviews have shown variable ptosis rates ranging from 9% to 41.7%. Overall, the side effects were generally non-severe and resolved over time.

This study reported a significant relationship between exotropia and first-dose response (p=0.026), indicating that exotropia was more likely in those who achieved orthotropia following the first injection. Additionally, a strong association was observed between the preoperative angle of deviation and the initial botulinum toxin dose (p <0.001), suggesting that clinicians adjusted dosing based on the severity of deviation. However, no significant associations were identified between strabismus type and first-dose response (p=0.787), nor between preoperative angle and the need for surgery (p=0.661). Similar findings were also reported in the literature, which suggests that while botulinum toxin dose decisions are influenced by deviation severity, outcomes like surgical need may be affected by other clinical factors [13,14].

This study revealed no association between ptosis and dose angle, which is in line with a study conducted by Niyaz et al. in Turkey [13]. The findings of the present study suggest that botulinum toxin is a safe and effective treatment option for esotropia, which might reduce the need for surgical intervention in select patients. This study provides local evidence that may support wider clinical use and guide future larger studies in Bahrain.

To the best of our knowledge, this study is the first of its kind in Bahrain to assess botulinum toxin outcomes in strabismus, particularly esotropia, providing important local data for clinical decision-making. The inclusion of all treated patients enhances the study's representativeness and relevance. However, the study has several limitations as well. The sample size was relatively small (n=31), limiting the generalizability and the power to detect smaller differences. All botulinum toxin injections were performed by the same consultant ophthalmologist, which ensured consistency in technique but may limit the generalizability of the findings to other surgeons or clinical settings. In addition, the study lacked a comparison group (e.g., patients undergoing surgery alone or untreated patients); hence, the descriptive design does not allow definitive conclusions regarding the relative efficacy of botulinum toxin injections compared to other treatment approaches. The injections were performed without EMG guidance, which may have led to variability in muscle targeting and may affect the outcomes as well.

## Conclusions

In summary, this study reveals that botulinum toxin is a promising treatment modality for strabismus, especially esotropia, in Bahrain, with most patients achieving ocular alignment after just one or two injections. The low rate of surgical intervention post-botulinum toxin treatment further supports its use as an effective alternative in the management of pediatric strabismus. Future prospective, multi-center studies with larger sample sizes and longer follow-up periods are recommended to validate these findings and optimize botulinum toxin treatment protocols. 
